# Advances in Nanomaterials for Photovoltaic Applications

**DOI:** 10.3390/nano12203702

**Published:** 2022-10-21

**Authors:** Vlad-Andrei Antohe

**Affiliations:** 1 Research and Development Center for Materials and Electronic & Optoelectronic Devices (MDEO), Faculty of Physics, University of Bucharest, 077125 Măgurele, Ilfov, Romania; vlad.antohe@fizica.unibuc.ro; 2 Institute of Condensed Matter and Nanosciences (IMCN), Université catholique de Louvain (UCLouvain), B-1348 Louvain-la-Neuve, Wallonie, Belgium; vlad.antohe@uclouvain.be

The development of novel nanomaterials became a subject of intensive research, due to high market needs for innovative applications in virtually all aspects of life. In particular, the evolution of photovoltaic (PV) devices encountered a great scientific progress in the last few years, mainly because solar power has great potential to cover society needs in the context of energy crisis the world is facing nowadays. In this case, the researchers’ efforts are especially directed lately into designing solar cell architectures with improved photo-conversion efficiency, while mainly employing environmentally-friendly materials and inherently maintaining low manufacturing costs. In this context, I am really delighted to observe the large diversity of topics chased by the authors in this Special Issue of *“Nanomaterials”*, dealing practically with all the four essential sub-systems of a PV cell, i.e., (i) photo-absorber component, (ii) “window” layer, (iii) electrons- and holes-transporter media, as well as (iv) charge collecting electrodes.

Basically, the most important component in a PV structure, whatever it is the generation it belongs to, is the main photo-absorber, as this material must be typically engineered to gather photons from as wide region of solar spectrum as possible. In this sense, there are several papers focused onto studying novel materials typically prepared by low-cost techniques, used as photoactive layers to design cheaper and more efficient PV devices. For instance, it is worth mentioning here the comprehensive review of S. Gedi et al. debating with the physical properties of a relatively new class of materials relying onto tin-based binary sulfides (Sn_x_S_y_), exclusively synthesized by simple chemical bath deposition (CBD) approaches [1]. Besides, as the authors present, these materials are ecologically-friendly, abundant on earth and they could be successfully employed not only in solar energy photo-conversion devices, but in other applications to generate “clean” energy, such as photocatalysis or thermoelectricity. Very good performances of the second generation’ solar cells based on copper indium gallium selenide (CIGS) thin films exclusively grown by electrochemical pathways are also demonstrated elsewhere [2], as well as the work on ultra-thin noncrystalline cadmium telluride (CdTe) films prepared by the novel blade coating technique that promises to offer a reliable solution for low-cost and large-scale fabrication of solar cells based on semiconducting nanocrystals [3]. Not in the least, good results were also obtained while testing other main absorber materials for PV cells like antimony selenoiodide (SbSeI) thin films [4], or by implementing specific innovative fabrication technologies such as engagement of aluminium arsenide (AlAs) capping layers onto indium arsenide/galium arsenide (InAs/GaAs) quantum dot (QD) structures for solar cells [5]. The latter strategy was proven to be effective on improving the photovoltaic efficiency, when compared to the reference traditional QD-based solar cells.

Subsequently, the properties of the “window” layer in a PV device are in most of the cases greatly responsible for the overall wideness of the photoactive region of the cell. Noteworthy, J. Lee et al. pointed out that the efficiency of the chalcogenide solar cells (i.e., specifically the CIGS-based) is highly-dependent on the quality of the “window” layer and especially on its thickness [6]. Although the application of such ultra-thin “window” layers is typically limited by the capabilities of the deposition method, the authors demonstrated that by replacing the commonly employed sputtering method with the atomic layer deposition (ALD) technique, ultra-thin zinc oxide (A-ZnO) layers (i.e., with a thickness of only 12 nm) could be achieved, featuring superior properties to act as “window” layers and to determine thus better photovoltaic performances when compared to those PV cells based on sputtered intrinsic zinc oxide (i-ZnO) “window” layers. According to the authors, the ALD process could be also advantageously engaged into the large-scale mass production of CIGS-based solar cells. In contrast, other researchers demonstrated that zinc selenide (ZnSe) thin films prepared by radio-frequency (RF) magnetron sputtering under optimized specific conditions could possess excellent physical properties to successfully act as environmentally-friendly “window” materials for solar cells, helping thus at diminishing the amount of cadmium (Cd) still commonly used for the second generation of solar cells relying on cadmium telluride (CdTe) as main absorber [7].

The transport of the photo-generated charge carriers towards the cell’s electrodes play also an important role in the overall photovoltaic response. For this reason, a great interest of the scientific community is dedicated to this process, as these materials should exhibit good compatibility with the neighbouring components of the cell and most importantly, they must feature an energetic band diagram structure with adequate LUMO (lowest unoccupied molecular orbital) and HOMO (highest occupied molecular orbital) levels that facilitate the transport of electrons and holes towards the cathode and anode electric contacts of the PV cell, respectively. In this sense, the thorough studies onto complexes based on titanium dioxide (TiO${}_{2}$) acting as electron transporter layer (ETL) materials must be acknowledged [8,9], as well as the exhaustive review on hole transporter layer (HTL) materials for organic PV cells by C. Anrango-Camacho et al. [10].

Ultimately, the collection of the photo-generated charge carriers substantially depend on the physical properties of the back- and top-electrodes of the cell. Obviously, at least one of these electrodes must combine a high electrical conductivity with an excellent transparency in the visible region of the electromagnetic spectrum. These two antagonistic properties are sometimes hard to reconcile, motivating the important work dedicated to the study of novel transparent conducting oxide (TCO) materials for PV cells. Herein, a new composite relying on indium-zinc-tin oxide (IZTO) was successfully engaged as a transparent electrode, the authors demonstrating its potential when used within construction of an ultra-flexible organic PV cell used for powering human wearable devices [11]. Other novel TCO materials with superior physical properties have been also studied by L. Hrostea et al. [12]. In this case, the authors deposited oxide/metal/oxide multi-layers as alternative to the well-studied indium-doped tin oxide (ITO), onto glass and especially plastic substrates, hence owning to a great potential for flexible photovoltaic devices.

It is obvious now that a great dedication of studying in details each building-block of the PV device is essential, as the morphological, compositional, structural, optical and photo-electrical properties of each of the constituting material have to be all-together well-harmonized in order to get the expected increase in the performance of a solar cell, whatever its type and generation.

## Data Availability

Data might be available from the authors of the cited papers.
